# Flow Injection-Inductively Coupled Plasma Spectrometry: A New Strategy for Ultratrace Analysis

**DOI:** 10.6028/jres.093.119

**Published:** 1988-06-01

**Authors:** C. W. McLeod, Y. Zhang, I. Cook, A. Cox, A. R. Date, Y. Y. Cheung

**Affiliations:** Department of Chemistry, Sheffield City Polytechnic, Sheffield, S1 1WB, U.K.; British Geological Survey, 64 Grays Inn Road, London, WC1X 8NG, U.K.

In the last decade few analytical techniques have made a greater impact in trace inorganic analysis than ICP emission spectrometry. Noteworthy features of the technique are well documented, but lack of sensitivity and susceptibility to spectral interferences have ensured that alternative techniques such as atomic absorption spectrometry remain competitive. The conventional approach to improving method sensitivity is to undertake relatively time-consuming sample pretreatment procedures such as ion exchange, solvent extraction, co-precipitation etc. With the advent of flow injection analysis [1] there has been a considerable interest in the development of on-line microcolumn concentration techniques for atomic spectrometry [2,3]. In the experiment relatively large volumes of sample are passed through a microcolumn in the flow injection (FI) manifold and retained analytes are subsequently eluted by injection of a small volume of eluent. Activated alumina offers a novel route for analyte preconcentration since it can function both as an anion and a cation exchanger depending upon solution pH. Under acidic and basic conditions alumina exhibits a high affinity for oxyanions and cations, respectively:

**Figure:**
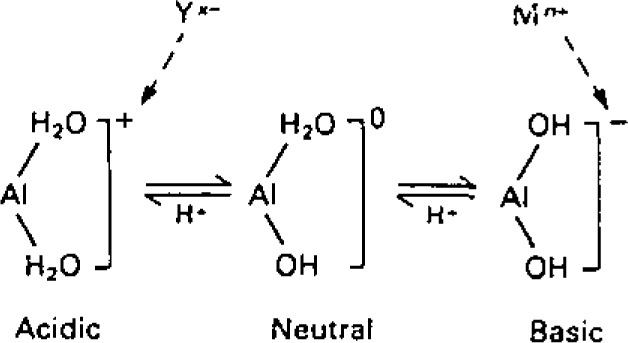


## On-line Trace Enrichment: Acidic Alumina

**Figure 1:**
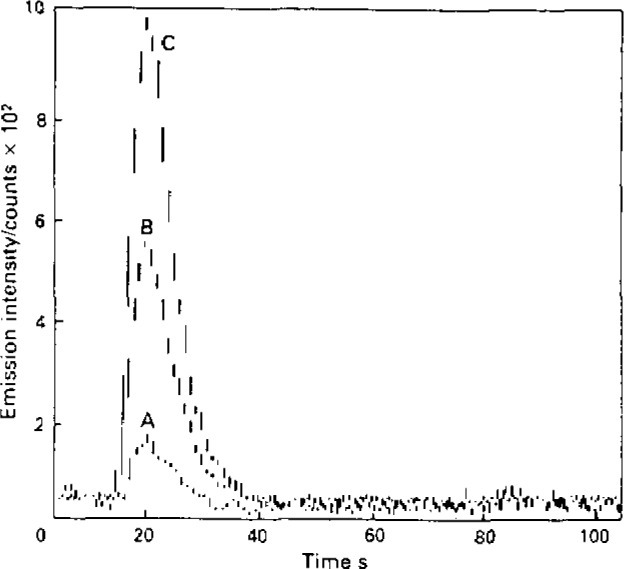
Effect of sampling volume on emission (267.7 nm)-time responses for Cr VI (20 μg/L): A, 200 μL; B, 1 niL; C, 2 mL. Elution, ammonium hydroxide (200 μL, 1 *M*).

The acidic form of alumina has a high affinity for a range of oxyanions and, as a result, FI-ICP methodology has been devised for the determination of phosphorus in steels, chromium VI in waters and sulphate in waters. The FI manifold consisted of a peristaltic pump, dual injection rotary valve, and a microcolumn (20 mm × 1.5 mm i.d.) of acidic alumina. In operation, samples were injected into the carrier stream and oxyanions were retained on the column whereas cationic species were unretained. An injection of ammonium or potassium hydroxide was then used to elute analyte into the ICP. Typical emission-time responses corresponding to on-line enrichment of chromate are presented in figure 1. The approach provided the basis for development of a rapid speciation scheme for inorganic chromium by virtue of the fact that the microcolumn has a high affinity for anionic chromium VI in contrast to that for chromium III. Thus on injection of samples containing the two chromium species, chromium III was not retained by the column and hence time-resolved emission signals corresponding to the two oxidation states were monitored. Analytical data for the determination of chromium III and chromium VI in a reference water (SRM 1643a, National Bureau of Standards) certified for total chromium content are given in table 1.

## On-line Trace Enrichment: Basic Alumina

**Figure 2:**
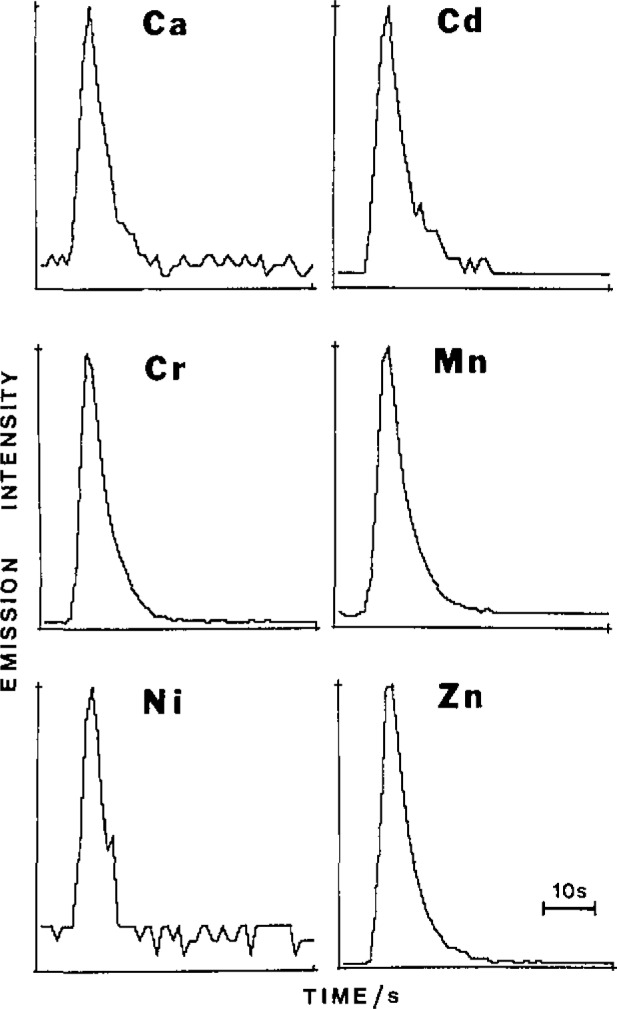
Multielement elution profiles: sample injection, multielement solution (250 µL, 0.1 µg/mL); elution, nitric acid (250 µL, 2 *M*)

**Figure 3:**
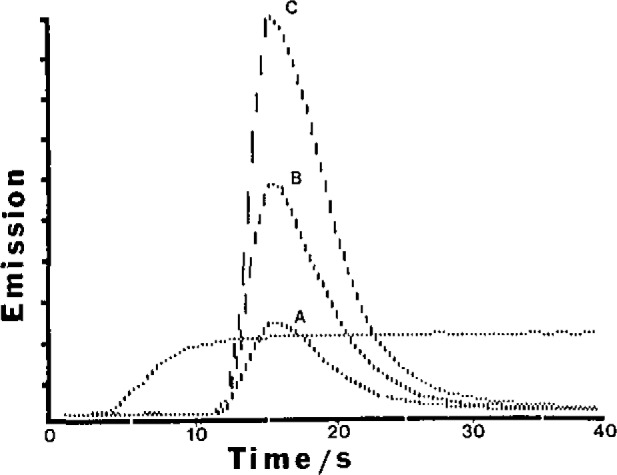
Effect of sampling time on emission (257.6 nm)-time responses for Mn (10 µg/L): A, 2 min; B, 5 min; C, 10 min. Sampling rate, 8 mL/min. Elution, nitric acid (250 µL, 2 *M*). Steady state response, conventional nébulisation (Mn 1 µg/L).

Recent studies have indicated that the FI manifold with basic alumina offers a relatively nonselective route for multielement preconcentration of divalent and trivalent cationic species over a wide pH range (pH 3–10). The emission-time responses for six elements given in Figure 2 reveal rapid exchange kinetics for the elution process. Also the same integration window (integration time of 17 s) is appropriate for monitoring the transient signals and this complies with the signal processing requirements of multichannel spectrometers. Minimal retention of the alkali elements, sodium, potassium and lithium was noted (except at pH > 10) and hence the technique offers a matrix isolation capability for those samples containing high concentrations of sodium and potassium. The same conclusion, however, does not apply to alkaline earth cations. Typical experimental conditions were: carrier stream, ammonium hydroxide (0.02 *M*); eluent, nitric acid (2*M*); samples buffered to pH 6 and containing tartaric acid (0.5 *M*) as complexing agent. Use of tartaric acid was found to maintain a high deposition efficiency over a wide pH range relative to results for simple aqueous solutions without complexing agent. In addition the breakthrough capacity of the column was extended for the former case and this enabled the use of extended sampling times (e.g., 10 min at 8 mL/min) to achieve relatively high preconcentration factors. This aspect is illustrated in figure 3, in the case of manganese, where it can be seen that a 10 min sampling time corresponded to a preconcentration factor of about 500. Similar results were realized for barium, calcium, cadmium, cobalt, chromium, copper, iron, magnesium, nickel, lead, and zinc. At the 10 µg/L level and for 2 min sampling (sampling rate 8 mL/min) relative standard deviations for the specified elements were in the range 0.8–2.0% (exceptions Pb 3.9%; Ni 4.3%), For an assessment of accuracy the procedure was applied to the determination of trace elements in a certified reference water (SLRS-1, National Research Council of Canada). The data presented in table 2 reveal some inaccuracies (particularly Cd, Co, and Pb) and this was considered to be due to contamination in sample preparation/processing.

## Conclusion

A FI system incorporating a microcolumn of activated alumina has been devised for rapid on-line preconcentration in ICP emission spectrometry. Preconcentration factors of at least 100-fold are realized for a wide range of cationic and anionic species at sampling times of several minutes. The FI experiment may be coupled to alternative techniques including flame atomic absorption, spectrometry and solution spectrophotometry and hence there is considerable scope for redeployment of such instrumentation for ultratrace analysis.

## Figures and Tables

**Table 1:** Analytical data (μg/L) for chromium III and chromium VI in water CRM (NBS 1643a)

Injection volume	Cr III	Cr VI
200 μL.	15.0±1.2	not detected
2 mL	14.8±1.0	1.96±0.32

**Table 2:** Analytical data (µg/L) for water CRM (NRCC SLRS-1)

	FI-ICP-ES^a^	Certificate
Cd	0.028	0.015±0.002
Co	0.058	0.043±0.010
Cu	3.10	3.58±0.30
Fe	33.3	31.5±2.1
Mn	1.30	1.77±0.23
Ni	1.10	1.07±0.06
Pb	0.70	0.106±0.011
Zn	1.23	1.34±0.20

a 3 min sampling time; in duplicate.
